# Contributions of preeclampsia to preterm delivery: a hypothetical interventional cohort study using incremental propensity scores

**DOI:** 10.1093/ije/dyag098

**Published:** 2026-07-09

**Authors:** Wen Wei Loh, Cande V Ananth

**Affiliations:** Department of Methodology and Statistics, Maastricht University, Maastricht, The Netherlands; Division of Epidemiology and Biostatistics, Department of Obstetrics, Gynecology, and Reproductive Sciences, Rutgers Robert Wood Johnson Medical School, New Brunswick, NJ, United States; Cardiovascular Institute of New Jersey, Rutgers Robert Wood Johnson Medical School, New Brunswick, NJ, United States; Department of Medicine, Rutgers Robert Wood Johnson Medical School, New Brunswick, NJ, United States; Department of Biostatistics and Epidemiology, Rutgers School of Public Health, Piscataway, NJ, United States; Environmental and Occupational Health Sciences Institute (EOHSI), Rutgers Robert Wood Johnson Medical School, New Brunswick, NJ, United States

**Keywords:** incremental propensity score intervention (IPSI), causal estimand, gestational hypertension, preeclampsia, preterm delivery, sensitivity analysis, unmeasured confounding

## Abstract

**Background:**

Preeclampsia remains a leading cause of preterm delivery (PTD). Conventional average causal effect estimands may not always be perfectly suited for assessing preeclampsia’s contributions because they rely on uniform counterfactual scenarios, such as everyone suffering from preeclampsia. A complementary approach is the incremental propensity score intervention (IPSI), which can address a more action-oriented causal inquiry: How would the population average risk of PTD change from current observed levels if each individual’s odds of preeclampsia were reduced by a given amount?

**Methods:**

We applied IPSI to two US population cohorts of singleton pregnancies, separated by two decades (1998–2002, 16.7 million; 2020–2023, 14.2 million), to evaluate the causal effects of gestational hypertension/preeclampsia (GHTN/PE) on PTD within each cohort. We developed and implemented a sensitivity analysis procedure using bias formulas to gauge the impact of potential unmeasured confounding.

**Results:**

Between 1998 and 2002, halving the individual odds of GHTN/PE would reduce the population average risk of PTD from observed [risk ratio (RR) = 0.969, 95% confidence interval (CI) 0.969–0.970]. Between 2020 and 2023, when GHTN/PE was more prevalent, halving the odds of preeclampsia would lead to an even greater reduction in average PTD risk (RR = 0.948, 95% CI 0.947–0.948). Sensitivity analyses demonstrated that these conclusions held under assumptions encoding relatively strong unmeasured confounding.

**Conclusions:**

The IPSI approach offers more nuanced understanding and clinically meaningful interpretations of causal effects, such as by how much partially reducing the odds of GHTN/PE, while allowing it to remain non-zero, can lower the average risk of PTD.

Key Messages

- Using the incremental propensity score intervention (IPSI) approach, we seek to answer the causal query: How would the average risk of preterm delivery change from current observed levels if we merely reduced each individual’s odds of preeclampsia while still allowing it to remain non-zero?
- In each of two birth cohorts separated by two decades, we found that modest reductions in the odds of gestational hypertension/preeclampsia as recorded in US vital records could already lower the average risk of preterm delivery, even when allowing for relatively strong unmeasured confounding.
- By applying IPSI, we gained unique insights into how practically achievable partial reductions in the odds of gestational hypertension/preeclampsia can lower the population average risk of preterm delivery relative to current observed levels in the US.

## Introduction

Preeclampsia is a leading cause of adverse perinatal outcomes, including preterm delivery (PTD) [1, 2] and perinatal mortality [3, 4]. Assessing preeclampsia’s effects often involves estimating average causal effects (ACEs) that compare counterfactual scenarios in which everyone experiences the same exposure level. While ACEs are essential for enhancing scientific understanding of causal relationships, they may not always be ideal for all causal queries [5]. First, relying solely on counterfactual scenarios precludes comparisons to observed, factual outcomes. Second, a practically feasible intervention that can achieve either scenario (e.g. eliminating or inducing preeclampsia across the entire population) may be less achievable.

We implement a complementary approach that permits more nuanced assessments of preeclampsia’s contributions to PTD: the incremental propensity score intervention (IPSI [6]). IPSI frames causal queries as: How would the currently observed population average outcome change by reducing the propensity score a given amount (while remaining greater than zero)? IPSI integrates real-world heterogeneity in the individual odds of exposure into its definition and considers hypothetical interventions aimed at reducing—without eliminating or mandating—the exposure. IPSI has recently been introduced to investigate the effects of diet on preeclampsia [7] and of job loss on self-injury mortality [8].

We apply IPSI to evaluate preeclampsia’s causal effects on PTD using two US population cohorts of singleton pregnancies two decades apart. IPSI is ideally suited for this causal endeavor because preeclampsia is not experienced uniformly: real-world interventions reduce the risk of preeclampsia by varying degrees, but do not eliminate it. It is unrealistic to eliminate preeclampsia because a certain portion of preeclampsia cases may be due to developmental errors in placental implantation, such as uteroplacental dysfunction, that are difficult or impossible to prevent [9–11].

To assess IPSI using risk ratios (RRs), we describe a causal RR as the ratio of the average IPSI outcome to the observed outcome. This RR addresses the causal query: How would the population average risk of PTD differ from currently observed levels if each individual’s odds of preeclampsia were reduced? Finally, as with most causal analyses using observational data, conclusions using IPSI remain susceptible to confounding bias. Because no sensitivity analyses specific to the IPSI paradigm are currently available, we developed a procedure for IPSI using established bias formulas for the ACE [12] to gauge the impact of unmeasured confounding.

## Methods

We conducted a secondary analysis of data from two US population cohorts of singleton pregnancies delivered at ≥20 weeks of gestation (16.7 million, 1998–2002; 14.2 million, 2020–2023). These data are derived from the US live birth and fetal death records assembled by the National Center for Health Statistics of the Centers for Disease Control and Prevention. No institutional review board approval was sought as these data are deidentified and publicly available. We contextualize the IPSI within each cohort spanning two decades because preeclampsia prevalence in the US increased between 1980 and 2010 [13], whereas PTD rates remained relatively stable due to improved obstetrical care.

Our exposure (*X*) was a broad classification, as recorded in US vital records, that included diagnoses of either hypertension only (gestational hypertension, defined as systolic blood pressure ≥140 mmHg and/or diastolic blood pressure ≥90 mmHg, diagnosed at ≥20 weeks of gestation) or hypertension with proteinuria (preeclampsia) [14]. We henceforth refer to this composite simply as “GHTN/PE.” Validation studies showed limited sensitivity for gestational hypertension (33% in New York City; 66% in Vermont) but high specificity (>99%) [15]. PTD was the binary outcome *Y* (=1 if gestational age < 37 completed weeks). Let $Y^{1}$ and $Y^{0}$ denote the potential PTD outcome with or without GHTN/PE, respectively. We maintained causal consistency (assumption 1 of Kennedy [6]) so that $Y=XY^{1}+(1-X)Y^{0}$ almost surely. Baseline covariates, jointly denoted by *C*, were maternal age (<15, 15–19,…, 40–44, and ≥45 years), live-born parity (primiparous, parity 2, and parity ≥3), maternal education (<9, 9–12, 13–16, and ≥17 years of schooling), race/ethnicity (White, Black, or other in 1998–2002; Non-Hispanic White, Non-Hispanic Black, Hispanic, or other in 2020–23), tobacco smoking during pregnancy (nonsmoker or smoker), marital status (married or single; only in 1998–2002), chronic hypertension, pre-pregnancy body mass index (BMI; only in 2020–23), pregestational diabetes mellitus (only in 2020–23), and calendar year of birth. We intentionally avoided stratifying by or adjusting for causal intermediaries between GHTN/PE and PTD (e.g. gestational age) to maintain the causal effect’s definition and avoid overadjustment bias [16–18].

### Incremental propensity score intervention

We briefly introduce IPSI [6]. Let π(C)=Pr(X=1|C) denote an individual’s current propensity score for GHTN/PE. An IPSI is characterized by a shift in π(C) parameterized by δ [5, 6]. Specifically, let $Q_{\delta }(C)$ denote the shifted propensity score. Then, δ describes the odds ratio between $Q_{\delta }(C)$ and π(C), so that solving for $Q_{\delta }(C)$ yields the functional form:


(1) $$Q_{\delta }(C)=\frac{\delta \pi (C)}{1+(\delta -1)\pi (C)}.$$

The ensuing IPSI average outcome under (1) for a given δ is then:


(2) $$E(Y^{Q_{\delta }})=\int \left[E(Y^{1}|C)Q_{\delta }(C)+E(Y^{0}|C)\{1-Q_{\delta }(C)\}\right]dF(C).$$

The causal quantity in (2) addresses the query: What is the average PTD risk after shifting each individual’s current propensity of GHTN/PE by δ through an intervention to $Q_{\delta }(C)$? The IPSI average outcome is assessed for a posited grid of values for δ encoding hypothetical interventions on GHTN/PE of different magnitudes, with δ=1 recovering the observed outcome E(Y). Relevant considerations for practically determining these values, whether a uniform value is necessary, and when the IPSI equals the ACE are addressed in the discussion. Details of IPSI, including estimation using the influence function and inference using confidence intervals (CIs) via the multiplier bootstrap, are explained elsewhere [5–7].

Substantive interest is often also in the relative change between the IPSI average outcome and the observed outcome (under δ=1). We consider the ratio of the average risk of PTD under a given δ<1 (representing lowered odds of GHTN/PE) to under δ=1 (at current levels), defined as $\text{RR}(\delta )=\frac{E(Y^{Q_{\delta }})}{E(Y)}$. Its asymptotic variance can be estimated through an application of the delta method:


(3) $$\mathrm{var}\left\{\text{RR}(\delta )\right\}=\mathrm{var}\left\{\frac{E(Y^{Q_{\delta }})}{E(Y)}\right\}=\frac{\mathrm{var}\{E(Y^{Q_{\delta }})\}}{E{\left(Y\right)}^{2}}-2\frac{E(Y^{Q_{\delta }})\mathrm{cov}\{E(Y^{Q_{\delta }}),E(Y)\}}{E{\left(Y\right)}^{3}}+\frac{E{\left(Y^{Q_{\delta }}\right)}^{2}\mathrm{var}\{E(Y)\}}{E{\left(Y\right)}^{4}}.$$

The covariance of the average IPSI and observed outcomes, $\mathrm{cov}\{E(Y^{Q_{\delta }}),E(Y)\}$, can be obtained using the covariance between the respective individual influence functions. An asymptotic Normal-based 100(1−α)% CI would then be $\hat{\text{RR}}(\delta )\pm z_{1-\alpha /2}\sqrt{\hat{\mathrm{var}}\left\{\text{RR}(\delta )\right\}}$, where $z_{1-\alpha /2}$ denotes the 100(1−α/2) percentile of a standard normal distribution. The percentile can be adjusted to account for *d* pairwise comparisons of posited δ values by replacing α with α/d using a Bonferroni correction.

We fitted a logistic regression model to estimate propensity scores, including all main effects and two-way interaction terms among the covariates. To predict potential outcomes, we fitted separate logistic regression models for each exposure subgroup, each including all main effects and two-way interaction terms among the covariates. This allowed for different exposure-specific coefficients and was equivalent to a single outcome model with all possible exposure-covariate(-covariate) interactions.

### Missing data

No data were missing for either GHTN/PE or PTD in the 1998–2002 cohort. There were no missing data for PTD in the 2020–23 cohort; however, approximately 0.2% had missing GHTN/PE status. A few baseline covariates had missing values in each cohort. To account for partial missingness, we applied multiple imputations with chained equations for each variable predicted by all other variables, using predictive mean matching [19], assuming data were missing at random. Each imputation was used to complete the dataset before estimating the IPSI average outcomes, and the estimates from twenty imputations were combined using Rubin’s rule. We did not impute covariates entirely missing in each cohort (pre-pregnancy BMI and pregestational diabetes mellitus in 1998–2002; marital status in 2020–23).

### Sensitivity analysis to unmeasured confounding for IPSI

Consistent estimation of IPSI causal effects in (2) assumes no unmeasured confounding (assumption 2 of Kennedy [6]):


(4) $$Y^{x}╨\;X|C;\;x=0,1.$$

We propose a sensitivity analysis procedure specific to IPSI for putative violations using bias correction formulas for the ACE [12]. Details are provided in Box 1.

However, there are two practical challenges specific to IPSI. First, it is computationally infeasible to recalculate IPSI estimates and CIs for all posited values of δ while holding the sensitivity parameters, whose cardinality grows exponentially with their granularity, fixed. Second, unlike other causal effect estimands [20, 21], it is practically impossible to visualize the IPSI average outcomes varying across δ and the sensitivity parameters simultaneously. Therefore, we simplify the assumptions following current methods for the ACE [12] to present sensitivity analyses for unmeasured confounding that systematically vary the sensitivity parameter values. Details are provided in the Supplementary Material.

## Results

GHTN/PE prevalence increased between the two cohorts. Of the 16.7 million singleton births between 1998 and 2002, 4.1% had GHTN/PE. Of the 14.2 million singleton births between 2020 and 2023, 9.3% had GHTN/PE. The distributions of maternal characteristics by GHTN/PE for each cohort are shown in Table 1. PTD risk remained relatively stable between the two cohorts (8.6% in 1998–2002; 9.0% in 2020–23). We first calculated the ACE. The average counterfactual PTD risk assuming no one had GHTN/PE was 8.1% (95% CI 8.0–8.1) in both cohorts, but the counterfactual risk assuming everyone had GHTN/PE was 21.2% (95% CI 21.1–21.3) in 1998–2002 and 19.4% (95% CI 19.3–19.5) in 2020–23. Therefore, the ACE (RR) was 2.63 (95% CI 2.62–2.65) and 2.42 (95% CI 2.41–2.44), respectively.

**Table 1:** Distribution of maternal characteristics in relation to GHTN/PE for all US singleton births in 1998–2002 and 2020–23.

	**1998–2002**						**2020–23**							
	**Total births**		**GHTN/PE**		**No GHTN/PE**		**Total births**		**GHTN/PE**		**No GHTN/PE**		**Missing**	
Variable	Number	%	Number	%	Number	%	Number	%	Number	%	Number	%	Number	%
Number of births	16 700 491	100.0	690 761	100.0	16 009 730	100.0	14 167 656	100.0	1 312 366	100.0	12 833 167	100.0	22 123	100.0
Maternal age (years)														
<15	37 119	0.2	2281	0.3	34 838	0.2	7185	0.1	684	0.1	6470	0.1	31	0.1
15–19	1985 242	11.9	94 145	13.6	1 891 097	11.8	584 355	4.1	55 291	4.2	527 892	4.1	1172	5.3
20–24	4 267 232	25.6	178 713	25.9	4 088 519	25.5	2 523 066	17.8	237 332	18.1	2 281 627	17.8	4107	18.6
25–29	4 496 141	26.9	184 215	26.7	4 311 926	26.9	3 948 825	27.9	362 084	27.6	3 580 548	27.9	6193	28.0
30–34	3 779 891	22.6	141 757	20.5	3 638 134	22.7	4 272 112	30.2	379 770	28.9	3 886 282	30.3	6060	27.4
35–39	1 773 763	10.6	71 646	10.4	1 702 117	10.6	2 291 980	16.2	216 785	16.5	2 071 614	16.1	3581	16.2
40–44	346 111	2.1	17 056	2.5	329 055	2.1	503 162	3.6	55 349	4.2	446 935	3.5	878	4.0
≥45	14 992	0.1	948	0.1	14 044	0.1	36 971	0.3	5071	0.4	31 799	0.2	101	0.5
Parity														
Primiparous	5 517 001	33.0	328 903	47.6	5 188 098	32.4	5 602 768	39.5	665 274	50.7	4 930 027	38.4	7467	33.8
2	4 878 472	29.2	170 161	24.6	4 708 311	29.4	4 409 354	31.1	335 127	25.5	4 069 478	31.7	4749	21.5
≥3	6 226 674	37.3	189 286	27.4	6 037 388	37.7	4 114 796	29.0	309 313	23.6	3 799 291	29.6	6192	28.0
Missing	78 344	0.5	2411	0.3	75 933	0.5	40 738	0.3	2652	0.2	34 371	0.3	3715	16.8
Maternal education (years)														
<9	825 515	4.9	25 398	3.7	800 117	5.0	447 446	3.2	27 423	2.1	418 937	3.3	1086	4.9
9–12	7 931 093	47.5	330 584	47.9	7 600 509	47.5	4 859 410	34.3	442 144	33.7	4 409 060	34.4	8206	37.1
13–16	6 184 809	37.0	269 135	39.0	5 915 674	37.0	6 764 810	47.7	663 450	50.6	6 093 570	47.5	7790	35.2
≥17	1 747 201	10.5	64 930	9.4	1 682 271	10.5	1 862 600	13.1	161 382	12.3	1 699 256	13.2	1962	8.9
Missing	11 873	0.1	714	0.1	11 159	0.1	233 390	1.6	17 967	1.4	212 344	1.7	3079	13.9
Race/ethnicity														
White	13 119 210	78.6	544 012	78.8	12 575 198	78.5	7 143 347	50.4	705 491	53.8	6 427 535	50.1	10 321	46.7
Black	2 772 887	16.6	122 879	17.8	2 650 008	16.6	1 981 809	14.0	219 139	16.7	1 759 122	13.7	3548	16.0
Hispanic							3 581 038	25.3	274 463	20.9	3 301 741	25.7	4834	21.9
Others	808 394	4.8	23 870	3.5	784 524	4.9	1 461 462	10.3	113 273	8.6	1 344 769	10.5	3420	15.5
Smoking during pregnancy														
Nonsmoker	14 358 515	86.0	606 082	87.7	13 752 433	85.9	13 353 170	94.3	1 230 933	93.8	12 105 364	94.3	16 873	76.3
Smoker	2 017 408	12.1	72 194	10.5	1 945 214	12.2	751 715	5.3	75 839	5.8	673 768	5.3	2108	9.5
Missing	324 568	1.9	12 485	1.8	312 083	1.9	62 771	0.4	5594	0.4	54 035	0.4	3142	14.2
Marital status														
Married	11 061 633	66.2	451 026	65.3	10 610 607	66.3								
Single	5 623 235	33.7	238 771	34.6	5 384 464	33.6								
Missing	15 623	0.1	964	0.1	14 659	0.1								
Chronic hypertension														
Absent	16 475 241	98.7	686 946	99.4	15 788 295	98.6	13 749 396	97.0	1 309 976	99.8	12 439 420	96.9		
Present	139 421	0.8	3815	0.6	135 606	0.8	396 137	2.8	2390	0.2	393 747	3.1		
Missing	85 829	0.5	0	0.0	85 829	0.5	22 123	0.2					22 123	100.0
Prepregnancy BMI (kg/m 2)														
<18.5							381 132	2.7	17 417	1.3	362 968	2.8	747	3.4
[18.5,25)							4 944 163	34.9	283 251	21.6	4 653 809	36.3	7103	32.1
[25,30)							4 020 969	28.4	346 604	26.4	3 669 253	28.6	5112	23.1
[30,35)							2 366 070	16.7	279 723	21.3	2 083 637	16.2	2710	12.2
[35,40)							1 227 296	8.7	182 474	13.9	1 043 552	8.1	1270	5.7
≥40							931 432	6.6	176 626	13.5	753 873	5.9	933	4.2
Missing							296 594	2.1	26 271	2.0	266 075	2.1	4248	19.2
Pregestational diabetes mellitus														
Absent							13 983 401	98.7	1 281 077	97.6	12 702 324	99.0		
Present							162 132	1.1	31 289	2.4	130 843	1.0		
Missing							22 123	0.2					22 123	100.0
Preterm delivery														
Term	15 265 657	91.4	544 693	78.9	14 720 964	92.0	12 891 446	91.0	1 062 110	80.9	11 813 346	92.1	15 990	72.3
Preterm	1 434 834	8.6	146 068	21.1	1 288 766	8.0	1 276 210	9.0	250 256	19.1	1 019 821	7.9	6133	27.7

**Figure 1:**
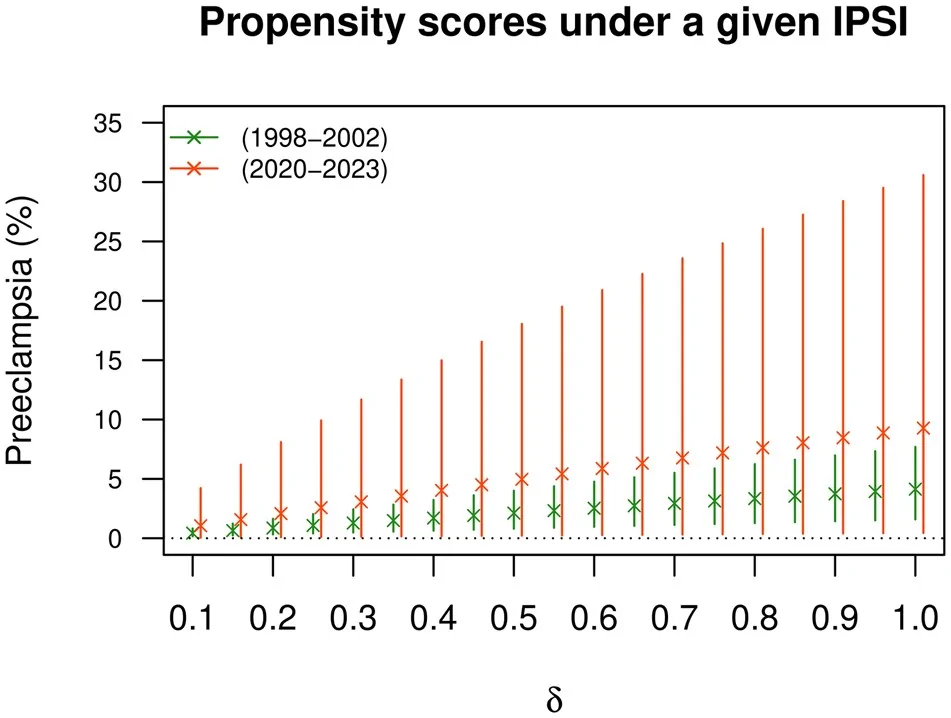
Distributions of propensity scores for GHTN/PE (%) under different IPSIs encoded by δ using US singleton births in 1998–2002 (green) and 2020–23 (red). The shift parameter δ encodes the odds ratio between the intended and naturally occurring odds of exposure. Each cross indicates the (marginal) average incremental propensity score under a given δ on the horizontal axis. Each vertical line represents the 0.5%- and 99.5%-tile of the propensity score distribution under a given δ. To improve visual clarity, the results for 2020–23 are shifted slightly to the right to avoid overlap.

We used IPSI to assess by how much the average PTD risk could be reduced by lowering the odds of GHTN/PE. Therefore, we focused on the values of δ≤1 and posited a uniformly spaced grid of δ=0.10,0.15,…,1.00 (i.e. odds of GHTN/PE reduced by 90%,85%,…,0%, respectively). The distribution of shifted propensity scores $Q_{\delta }(C)$ for each δ value within each cohort is plotted in Fig. 1. An IPSI that halves the odds of GHTN/PE (δ=0.5) reduced the average GHTN/PE risk (4.1% to 2.1% in 1998–2002; 9.3% to 5.0% in 2020–23). Hence, even for individuals at low risk of GHTN/PE (with smaller propensity scores), it remains feasible to further lower their risk. The propensity scores were more variable in the later cohort because the fitted model had higher predictive accuracy (C-statistic = 52% in 1998–2002, versus 67% in 2020–23). The absolute mean difference in covariates (including product interaction terms), reweighted by inverse propensity scores, was no larger than 0.006 in 1998–2002 and 0.008 in 2020–23, indicating measured covariate balance.

**Figure 2:**
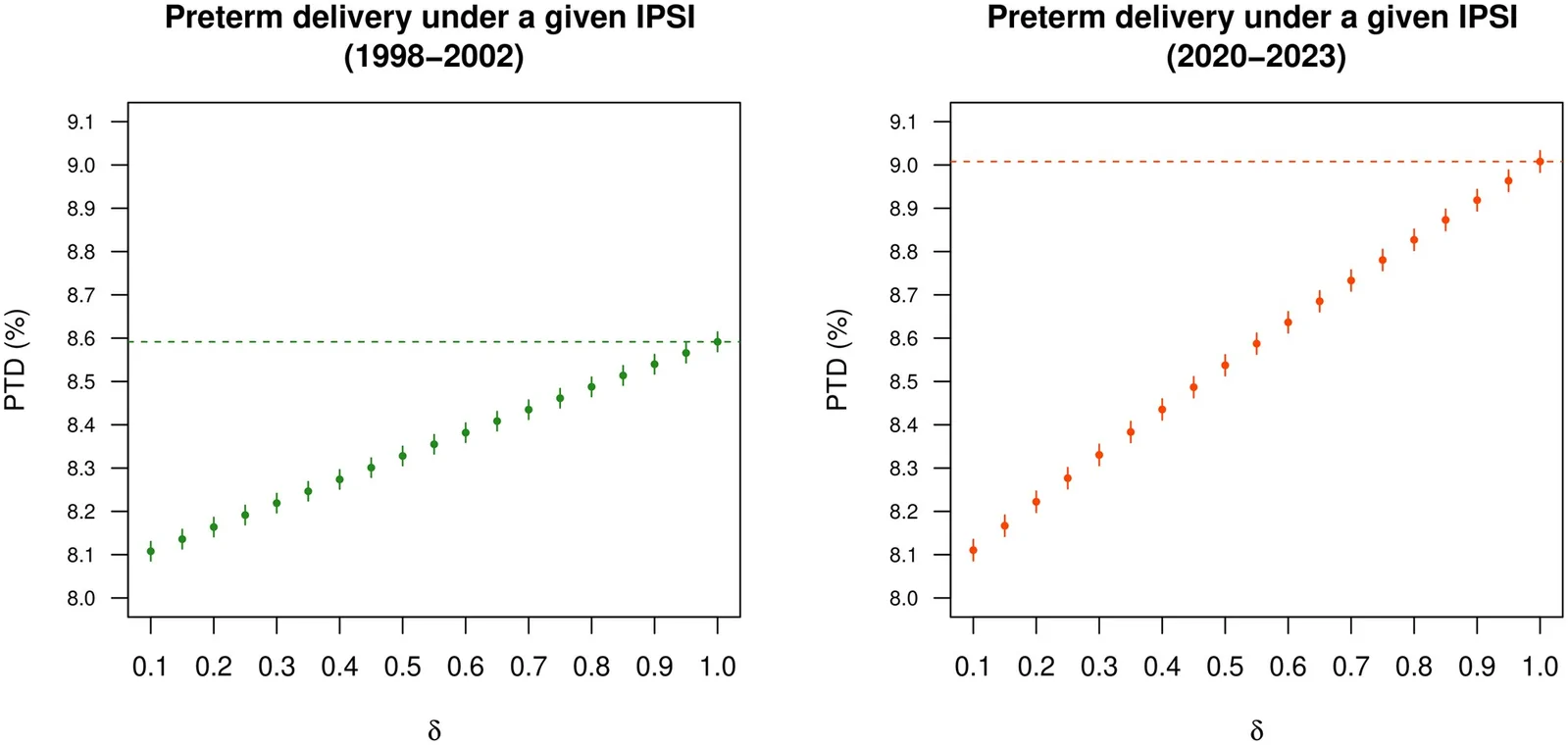
Estimates of the IPSI average PTD (%) under different IPSIs encoded by δ using US singleton births in 1998–2002 (left; green) and 2020–23 (right; red). The shift parameter δ encodes the odds ratio between the intended and naturally occurring odds of exposure. Each circle corresponds to a point estimate, and each vertical line corresponds to the 95% CI, under a given value of δ on the horizontal axis. Each vertical dotted line corresponds to a value of δ based on external real-world evidence. The horizontal broken line corresponds to the average observed risk of PTD under δ=1.

We can draw three conclusions from the IPSI results plotted in Fig. 2. First, as expected, when δ=1, the estimated average PTD equaled the observed sample mean (indicated by a horizontal broken line). Second, even modestly lowering the odds of GHTN/PE could lead to a commensurate decrease in the estimated average PTD risk. Between 1998 and 2002, halving the odds of GHTN/PE (δ=0.5) would lower the average PTD risk from the observed 8.59% (95% CI 8.57–8.67) to 8.33% (95% CI 8.31–8.35), with RR(0.5)=0.969 (95% CI 0.969–0.970). Between 2020 and 2023, halving the odds of GHTN/PE would lower the average PTD risk from the observed 9.01% (95% CI 8.98–9.03) to 8.54% (95% CI 8.51–8.56), with RR(0.5)= 0.948 (95% CI 0.947–0.948). The larger IPSI (RR) in the later cohort, using the same δ=0.5, was expected because GHTN/PE prevalence was higher while average PTD risk remained relatively stable; it did not, on its own, indicate a stronger causal effect of GHTN/PE on PTD. For the same reason, the ACE (RR) was weaker in the later cohort.

**Figure 3:**
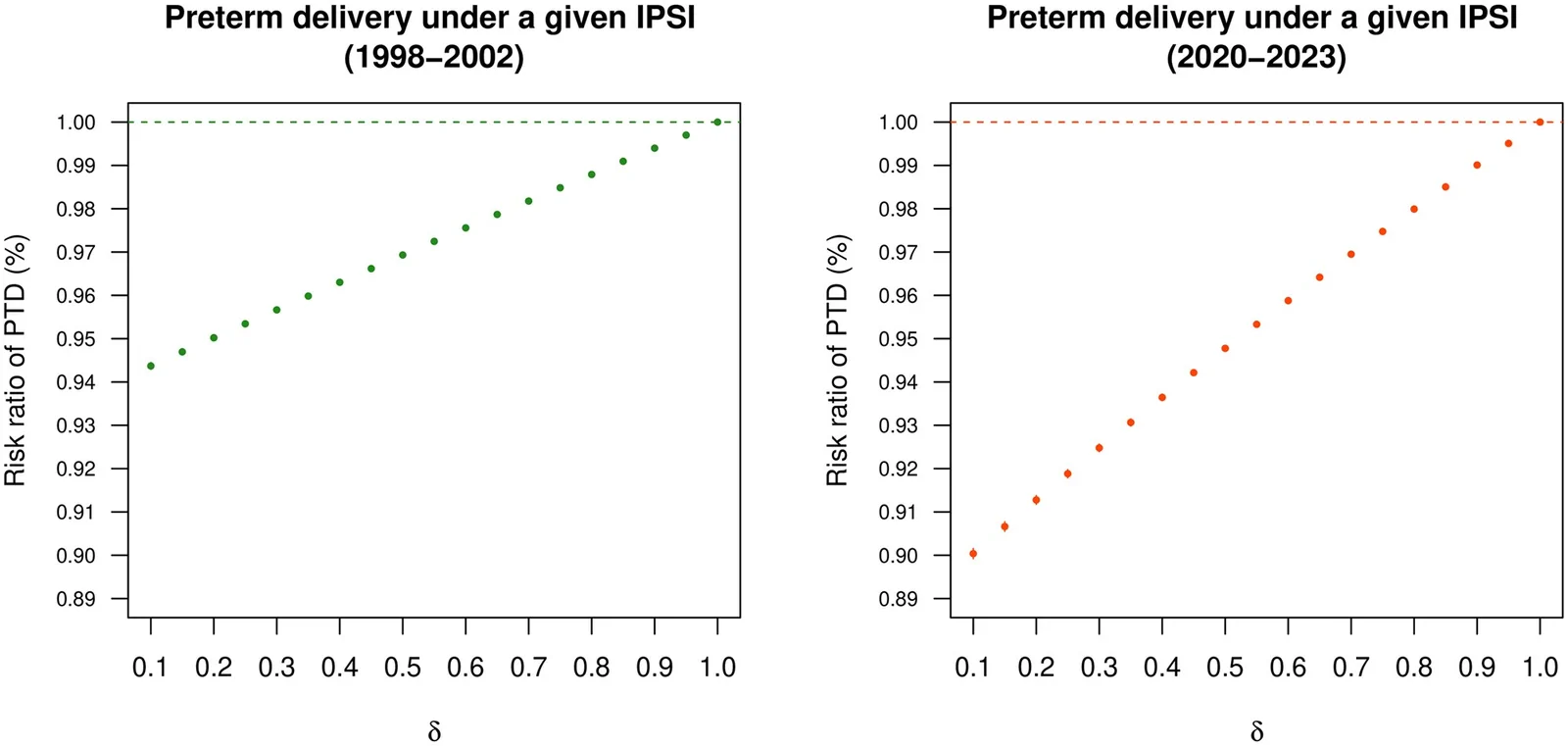
Estimates of the risk ratio contrasting the IPSI average PTD under each posited value of δ relative to the observed risk (δ=1) using US singleton births in 1998–2002 (left; green) and 2020–23 (right; red). The shift parameter δ encodes the odds ratio between the intended and naturally occurring odds of exposure. Each circle corresponds to a point estimate, and each vertical line corresponds to the 95% CI, under a given value of δ on the horizontal axis. The horizontal broken line indicates no difference.

Third, in both cohorts, suppressing the odds of GHTN/PE by 90% (δ=0.1) would potentially lead to almost the same reduced average PTD risk of 8.11% (95% CI 8.09–8.13). However, as shown in Fig. 3, the relative reduction in the later cohort [RR(0.1) = 0.900 (95% CI 0.899–0.902)] was larger than in the earlier cohort [RR(0.1) = 0.944 (95% CI 0.943–0.945)].

### BMI as a risk factor for preeclampsia and preterm delivery

BMI influences the risks of preeclampsia. However, because BMI was recorded only for the 2020–23 cohort, we examined its impact by deliberately excluding BMI and repeating the analysis for that cohort. First, excluding BMI resulted in smaller estimated propensity scores on average only among those with GHTN/PE (see Supplementary Material for Supplementary Fig. S1). Second, the predictive accuracy of the outcome models remained approximately the same regardless of whether BMI was included. The C-statistics for the separate models predicting PTD among GHTN/PE and no GHTN/PE births were 0.50 and 0.52, respectively, with BMI; after excluding BMI, the C-statistics remained unchanged (0.49 and 0.53, respectively). Finally, IPSI estimates without BMI were almost identical to those with BMI; the gradient of the estimates as δ decreased was only very slightly steeper for smaller δ values when excluding BMI (see Supplementary Material for Supplementary Fig. S2).

### Subgroup analyses

**Figure 4:**
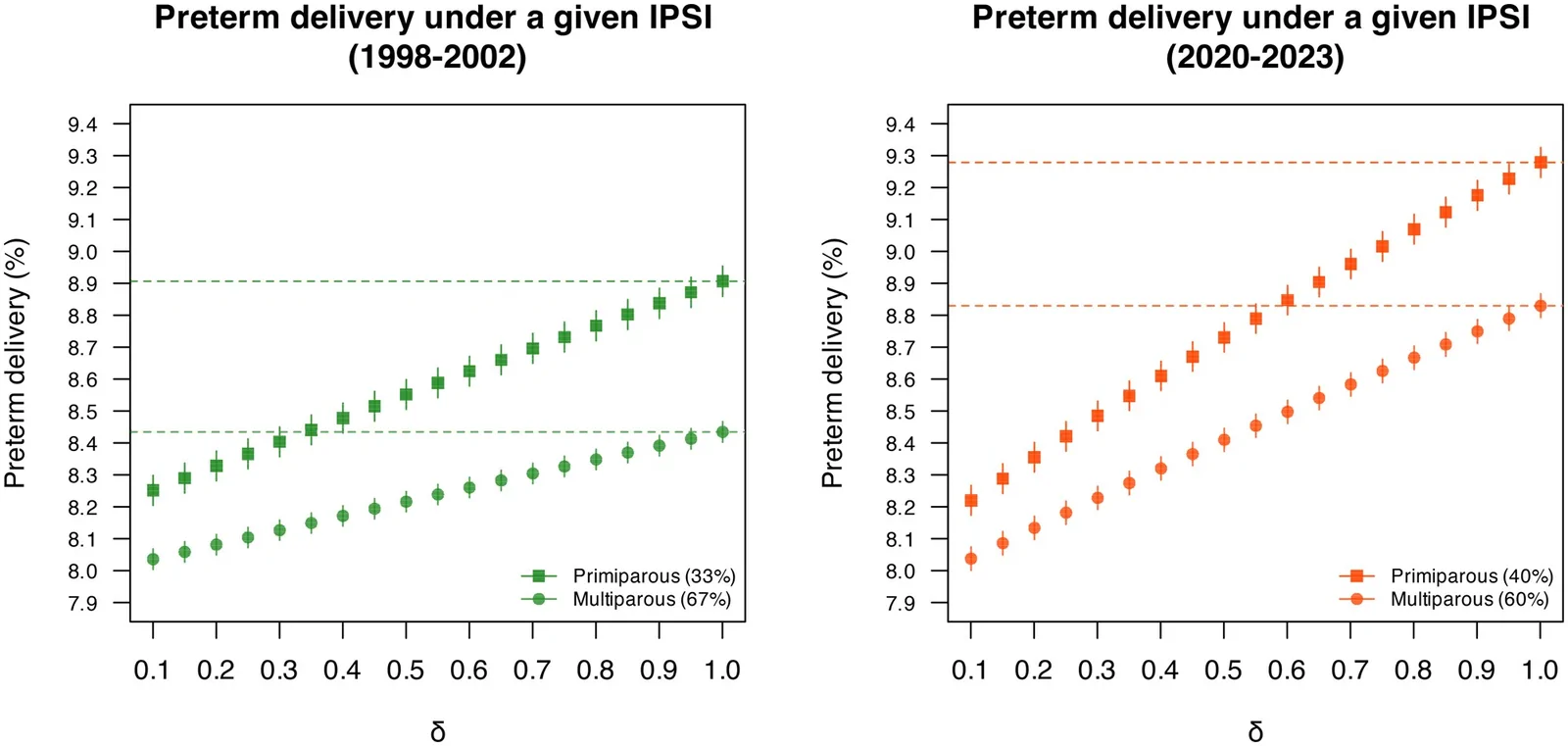
Subgroup-specific estimates of the IPSI average PTD (%) under different IPSIs encoded by δ using US singleton births stratified by parity. The left (green) and right (red) columns display the 1998–2002 and 2020–23 cohorts, respectively. Explanations of how to interpret each panel are provided in the caption of Fig. 2.

**Figure 5:**
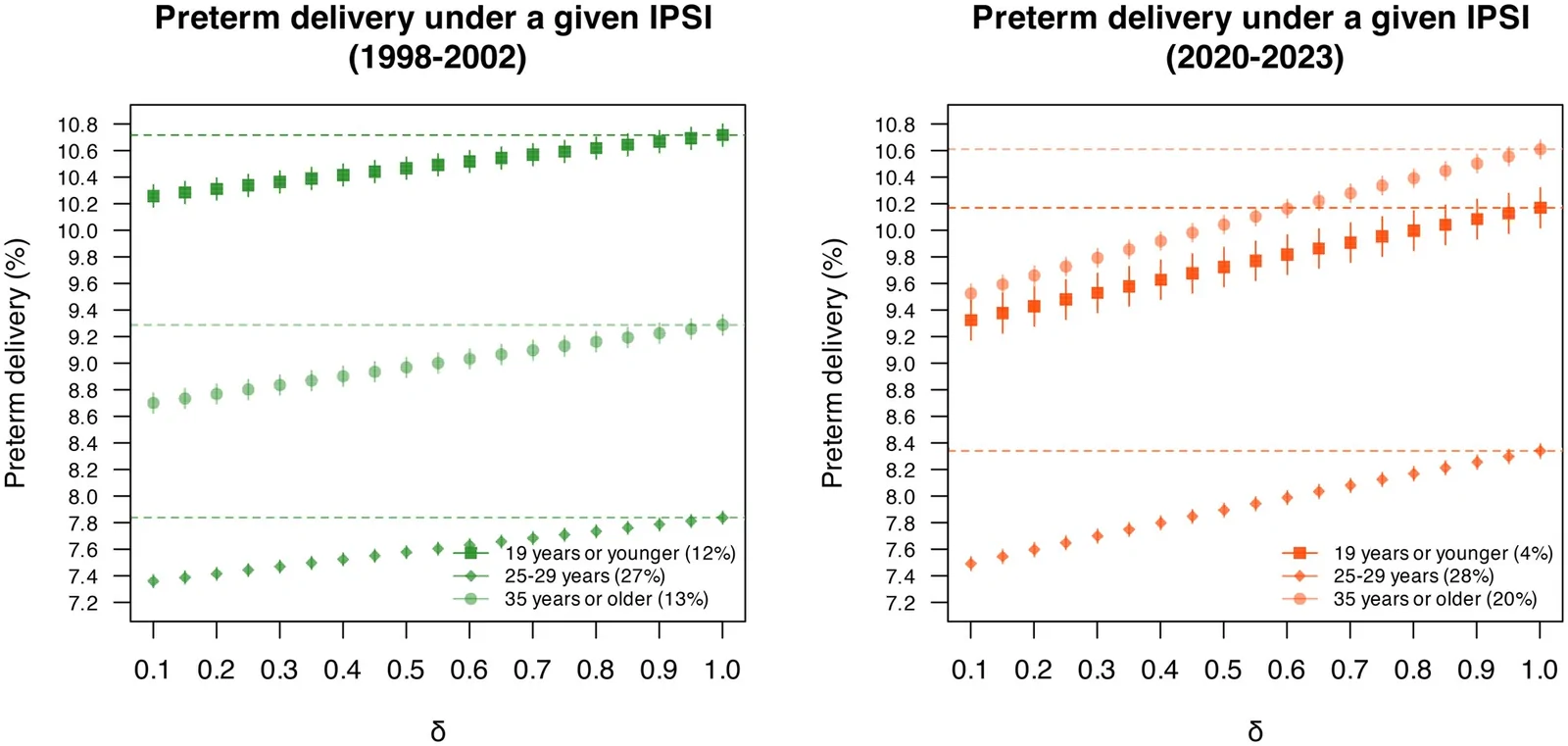
Subgroup-specific estimates of the IPSI average PTD (%) under different IPSIs encoded by δ using US singleton births stratified by maternal age group. The left (green) and right (red) columns display the 1998–2002 and 2020–23 cohorts, respectively. Explanations of how to interpret each panel are provided in the caption of Fig. 2.

We estimated subgroup effects by stratifying on parity or maternal age. The results in Fig. 4 indicate that primiparous births have a higher average PTD risk than multiparous births. The results in Fig. 5 indicate that the average PTD risk was highest among individuals ≤19 years old in the earlier cohort, but highest among those ≥35 years old in the later cohort.

### Unmeasured confounding bias

Using our proposed procedure, we undertook a sensitivity analysis of unmeasured confounding. We considered four distinct data-driven possibilities for the sensitivity parameter values. The results, plotted in Supplementary Figs S3 and S4, suggested that, across all four scenarios, we could maintain the conclusion that lowering the odds of GHTN/PE, even by a modest amount, would reduce average PTD risk.

## Discussion

### Principal findings

We sought to use IPSI to answer the causal query: How would the average PTD risk in the US change from current observed levels if we could partially reduce each individual’s odds of GHTN/PE? We demonstrated that halving the odds of GHTN/PE in 1998–2002 sufficed to lower PTD risk in the US population by RR(0.5) = 0.969. In a more contemporary cohort (2020–23) with almost twice the GHTN/PE prevalence of 1998–2002, while PTD risk remained relatively stable, applying the same hypothetical intervention would yield a larger reduction of RR(0.5) = 0.948. These counterfactual shifts in PTD from hypothetically intervening on GHTN/PE remained robust to certain violations of unmeasured confounding.

We conducted cohort-specific analyses based on identification assumptions within each cohort. No cross-cohort comparisons were formally tested because discrepancies between cohorts (e.g. changes in ascertaining GHTN/PE, different covariate adjustment sets, or coding) can affect estimates and undermine comparability. Hence, differences between cohorts should be interpreted cautiously rather than definitively as evolving effects.

### Comparison of IPSI causal estimands with other causal effects

We briefly compare the IPSI and ACE in Table 2. Importantly, while the ACE permits heterogeneous hypothetical interventions, they must produce the same exact endpoint of a uniform counterfactual scenario (e.g. eliminating or inducing GHTN/PE) across the entire population. This can lead to unrealistic promises about the causal effect because there may be no feasible intervention that can achieve either scenario [22, 23]. Hence, it is possible for a non-null ACE, whereas the IPSI does not achieve this because the shifts in individual δ are too small. We formally compare the IPSI with the (conditional) ACE, the average effect of treatment among the treated (ATT), and the average effect of treatment among the control/untreated (ATC/ATU) in the Supplementary Material.

**Table 2:** Comparison of the IPSI causal effect and the ACE.

	IPSI causal effect	ACE
Definition using potential outcomes	$\frac{E(Y^{Q_{\delta }})}{E(Y)}$	$\frac{E(Y^{1})}{E(Y^{0})}$
What is the change in the population average outcome …	…from the status quo if current observed exposure odds were shifted by δ?	…if everyone vs. no one was counterfactually exposed?
Expected change in the outcome brought about by …	…a hypothetical partial reduction in exposure odds vis-à-vis observed levels.	…comparing the hypothetical scenarios in which everyone vs. no one was counterfactually exposed
Multiplying each individual’s current odds of exposure by a factor …	…of δ, as defined in (1), that results in shifted propensity scores that vary across individuals.	…that results in a shifted propensity score in (1) exactly equal to either zero or one uniformly for everyone, regardless of their current propensity score.
Type of hypothetical intervention	Stochastic and dynamic	Deterministic and static
Comment	Shift parameter δ need not be uniform; it can be individual-specific based on subject matter knowledge	Corresponds to comparing IPSI causal effects where δ=∞ vs. δ=0, respectively.

In this paper, we considered IPSIs that shift the propensity score uniformly by δ for all individuals, but still allow the ensuing shifted propensity scores to vary across individuals. In practice, δ need not be identical for everyone. For example, one can postulate an IPSI that alters the odds of GHTN/PE differently depending on an individual’s preexisting PTD risk. Hence, researchers can leverage subject-matter knowledge to flexibly conceive IPSIs with fine-grained δ values.

### External real-world interventions have been shown to reduce preeclampsia risk

Preeclampsia complicates about 7%–10% of all pregnancies, but it confers disproportionately increased risks of PTD and other adverse perinatal outcomes. For instance, preeclampsia remains one of the chief indications for clinician-indicated PTD [1, 2] and a strong risk factor for spontaneous PTD [24]. Substantial efforts through prophylactic antiplatelet clinical interventions have been undertaken to reduce preeclampsia incidence. A meta-analysis [25] of 10 studies (18 911 subjects) showed that low-dose aspirin (81 mg/day) could achieve a reduction of 30% in the general population (RR 0.70, 95% CI 0.52–0.95). Furthermore, different strategies have been developed to prevent preeclampsia, depending on whether it is early-onset, which has a placental origin, or late-onset, which is associated with metabolic syndrome [26]. Given this evidence, hypothetical interventions to reduce but not entirely eliminate the risk of preeclampsia seem practically achievable and clinically justified.

### Obesity and preeclampsia risk

Pre-pregnancy overweight or obesity is a risk factor for preeclampsia [27]. Female obesity rates have increased from 33.1% in 2001–02 to 41.4% in 2017–18 in the US [28]. These findings prompted us to hypothesize that the increase in preeclampsia prevalence between the cohorts due to obesity may provide insights into whether a hypothetical preeclampsia intervention affects PTD. However, our fitted outcome models showed that BMI was a relatively weak predictor of PTD, alongside other covariates, to the extent that excluding BMI had a negligible impact on our results using IPSI.

### Limitations and future directions

Our application of IPSI is motivated by how IPSI enables quantification of GHTN/PE’s contribution to PTD on a continuum, such as by how much modest reductions in the individual odds of GHTN/PE will decrease average PTD risk. However, another motivation for using IPSI is that, when no unmeasured confounding (4) holds, positivity [29, 30] is unnecessary for consistent estimation [5–7]. While IPSI avoids the need to empirically diagnose violations of positivity, this does not preclude researchers from assessing or diagnosing putative violations in practice. For example, we found little evidence of a glaring lack of overlap in the propensity score distributions between GHTN/PE and no GHTN/PE in either cohort (see Supplementary Material for Supplementary Fig. S1).

We estimated propensity scores using logistic regression models with multiple interaction terms (the fitted model coefficients are in the Supplementary Material). Incorrect model specification can have a greater impact on IPSI estimates than on conventional doubly robust estimators of the ACE [7]. Machine learning algorithms can mitigate the risks of such biases [31]. IPSI estimators are based on efficient influence functions that enable these methods to model the propensity score and outcome [32].

## Conclusion

We argue that IPSI is better suited than ACE for assessing the nuanced causal contributions of preeclampsia in real-world, naturalistic settings with non-uniform exposure propensities. We developed and applied bias formulas for IPSI to gauge the impact of putative unmeasured confounding. IPSI is ideally suited to support the development of future interventions to reduce preeclampsia, while the methodology itself has far-reaching applications that could inform diverse public health priorities if widely applied to other obstetrical complications.

## Ethics approval

No institutional review board approval was sought as the data are deidentified and publicly available.

## Supplementary Material

dyag098_Supplementary_Data

## Data Availability

The data are publicly available and derived from the United States live birth and fetal death records assembled by the National Center for Health Statistics of the Centers for Disease Control and Prevention.

## Use of artificial intelligence (AI) tools

When preparing the revisions, AI-assisted tools (Grammarly and ChatGPT 5.2) were used to check spelling and grammar and to improve the readability of specific, selected statements. All text was reviewed, edited, and verified by the authors, who take full responsibility for the content of this final version.

Box 1. Sensitivity analysis assumptions and procedure Let *U* denote an unmeasured (set of) confounder(s) of *X* and *Y* so that adjusting for *C* and *U* jointly suffices to eliminate confounding bias; i.e.

(5)
$$Y^{x}╨\;X|C,U;\;x=0,1.$$

Let $\gamma_{\mathrm{xcu}}=\frac{E(Y|X=x,C=c,U=u)}{E(Y|X=x,C=c,U=0)}$ denote the conditional risk ratio for U=u relative to a reference level U=0. [29] The conditional average potential outcome $E(Y^{x}|C)$ can then be recovered from an estimator $\hat{E}(Y^{x}|C)$ that does not adjust for *U* using the closed-form expression for the bias factor $r_{x}(C)$ (on the risk ratio scale; Theorem 3 in VanderWeele and Arah [12]):

(6)
$$E(Y^{x}|C)=\frac{\hat{E}(Y^{x}|C)}{r_{x}(C)},\;r_{x}(c)=\frac{\sum_{u}\gamma_{\mathrm{xcu}}\mathrm{Pr}(U=u|X=x,C=c)}{\sum_{u}\gamma_{\mathrm{xcu}}\mathrm{Pr}(U=u|C=c)}.$$

Hence, adjusting for *U* when *U* and *X* are conditionally independent given *C* is unnecessary because Pr(U=u|X=x,C=c)=Pr(U=u|C=c) for all U=u so $r_{x}(c)=1$ for all C=c. This general expression allows for binary or multi-valued *U*, comprising either a single variable or a set of variables. A crucial distinction between the average causal effect (ACE) estimand and incremental propensity score intervention (IPSI) average outcome in (2) is that, under no unmeasured confounding, the positivity assumption [25, 32] is sufficient for identifying the former but unnecessary for the latter. The positivity assumption contingent on no unmeasured confounding in (4) is:

(7)
Pr(X=x|C=c)>0 for all x,c.

However, when there is unmeasured confounding so that (4) is violated, a sensitivity analysis using the bias factor approach then relies on the following version of positivity to ensure $r_{x}(C)$ is non-zero:

(8)
Pr(X=x|C=c,U=0)>0 for all x,c.

Positivity in (8) is also required to ensure $\gamma_{\mathrm{xcu}}$ takes on finite values, i.e.

(9)
Pr(Y=1|X=x,C=c,U=0)>0 for all x,c.

The reference level U=0 in defining $\gamma_{\mathrm{xcu}}$ must thus satisfy both (8) and (9). We elaborate on the technical details and implications of (8) and (9) in the Supplementary Material. Hence, when assuming no unmeasured confounding, positivity (given observed covariates) is unnecessary for IPSI, whereas allowing for unmeasured confounding introduces positivity (given observed and unobserved covariates) as a requirement to use these bias factors for a sensitivity analysis. A sensitivity analysis procedure, therefore, proceeds by fixing values for the joint sensitivity parameters $\gamma_{\mathrm{xcu}}$ and Pr(U=u|X=x,C=c), then calculating the bias factors $r_{x}(C),x=0,1$, for each individual with observed *C*. These bias factors are then applied to $\hat{E}(Y^{x}|C)$, the predicted potential outcomes using only *C*, to recover the adjusted potential outcomes accounting for *U*, e.g. $\tilde{E}(Y^{x}|C)=\hat{E}(Y^{x}|C)/r_{x}(C)$. The bias factors are also applied to $\hat{\pi }(C)$, the propensity score estimates using only *C*, to recover the adjusted propensity score, e.g. $\tilde{\pi }(C)=r_{1}(C)\hat{\pi }(C)$. The ensuing IPSI average outcome in (2), having accounted for unmeasured *U* under the fixed values of the sensitivity parameters, can be recalculated using $\tilde{\pi }(C)$ and $\tilde{E}(Y^{x}|C),x=0,1$.
