# Supplementary material for: Contributions of preeclampsia to preterm delivery: a hypothetical interventional cohort study using incremental propensity scores
Source: Int J Epidemiol. 2026 Jul 9;55(4):dyag098. doi: 10.1093/ije/dyag098 (PMC13350943; doi:10.1093/ije/dyag098)
Supplement: dyag098_Supplementary_Data [file dyag098_supplementary_data.zip › ije-2025-06-1031-File009.pdf]

Supplementary material for ‘Contributions of Preeclampsia to Preterm Delivery: A Hypothetical Interventional Cohort Study Using Incremental Propensity Scores’

Wen Wei Loh and Cande V. Ananth

May 27, 2026

Contents

A Comparisons of IPSI causal estimands with other causal effects **3**

B Sensitivity analysis for IPSI using bias correction factors **8**

B.1 Assumptions for using bias factors 9

B.2 Practical implementation 10

A Comparisons of IPSI causal estimands with other causal effects

In this section, we describe how the IPSI causal estimands are equivalent to other widely-used average causal effect estimands: the Average Causal Effect (ACE), the conditional ACE, the Average Effect of Treatment among the Control or Untreated (ATC/ATU), and the Average Effect of Treatment among the Treated (ATT). Recall that the average IPSI outcome (2) in the main text for a given $Q_\delta(C)$ is defined as:

$$\mathrm{E}(Y^{Q_\delta}) = \int \left[\mathrm{E}(Y^1|C)Q_\delta(C) + \mathrm{E}(Y^0|C)\{1 - Q_\delta(C)\}\right] dF(C).$$

Because setting $\delta = \infty$ yields the IPSI $Q_{\delta=\infty}(C) = 1$ and setting $\delta = 0$ yields the IPSI $Q_{\delta=0}(C) = 0$, we can express the ACE as the difference between these two IPSI average outcomes as follows:

$$\begin{aligned}
\mathrm{ACE} &= \mathrm{E}(Y^1 - Y^0) \\
&= \int \mathrm{E}(Y^1 - Y^0|C)\, dF(C) \\
&= \int \mathrm{E}(Y^1 - Y^0|C)\, \{Q_{\delta=\infty}(C) - Q_{\delta=0}(C)\}\ dF(C) \\
&= \int \mathrm{E}(Y^1|C)Q_{\delta=\infty}(C)\, dF(C) - \int \mathrm{E}(Y^0|C)\{1 - Q_{\delta=0}(C)\}\, dF(C) \\
&= \mathrm{E}(Y^{Q_{\delta=\infty}}) - \mathrm{E}(Y^{Q_{\delta=0}}).
\end{aligned}$$

Therefore, a null ACE implies that the IPSI effects will also be null across all specified δ values, so that $\mathrm{E}(Y^{Q_{\delta=\infty}}) = \mathrm{E}(Y^{Q_{\delta=0}}) = \mathrm{E}(Y)$, where the last term corresponds to $\delta = 1$. In contrast, a non-null ACE does not necessarily imply non-null IPSI effects for all δ values, because the shifts in individual δ may yield changes in the average outcome from the observed (under $\delta = 1$) that are too small.

We can further express the IPSI causal effect in terms of conditional effects (CACE). First, the average observed outcome is:

$$\begin{aligned}
\mathrm{E}(Y) &= \int \mathrm{E}[Y^1X + Y^0(1 - X)|C]\, dF(C) \\
&= \int \left[\mathrm{E}(Y^1|X = 1, C)\pi(C) + \mathrm{E}(Y^0|X = 0, C)\{1 - \pi(C)\}\right] dF(C) \\
&= \int \Big[\mathrm{E}(Y^1|X = 1, C)\pi(C) + \mathrm{E}(Y^1|X = 0, C)\{1 - \pi(C)\} \\
&\qquad - \mathrm{E}(Y^1|X = 0, C)\{1 - \pi(C)\} + \mathrm{E}(Y^0|X = 0, C)\{1 - \pi(C)\}\Big]\ dF(C) \\
&= \int \left[\mathrm{E}(Y^1|C) - \mathrm{E}(Y^1 - Y^0|X = 0, C)\{1 - \pi(C)\}\right] dF(C).
\end{aligned}$$

The first equality follows from causal or counterfactual consistency so that $Y = Y^1X + Y^0(1 - X)$, and the

second and last equalities follow from the law of total expectation.

The IPSI causal effect is thus:

$$
\begin{aligned}
&\mathrm{E}(Y^{Q_\delta}) - \mathrm{E}(Y) \\
&= \int \left[\mathrm{E}(Y^1 - Y^0|C)Q_\delta(C) + \mathrm{E}(Y^0|C)\right]\, dF(C) - \int \left[\mathrm{E}(Y^1|C) - \mathrm{E}(Y^1 - Y^0|X=0, C)\{1-\pi(C)\}\right]\, dF(C) \\
&= \int \left[\mathrm{E}(Y^1 - Y^0|C)\{Q_\delta(C) - 1\} + \mathrm{E}(Y^1 - Y^0|X=0, C)\{1-\pi(C)\}\right]\, dF(C) \\
&= \int \left[\left[\mathrm{E}(Y^1 - Y^0|X=1, C)\pi(C) + \mathrm{E}(Y^1 - Y^0|X=0, C)\{1-\pi(C)\}\right]\{Q_\delta(C) - 1\}\right. \\
&\quad \left. + \,\mathrm{E}(Y^1 - Y^0|X=0, C)\{1-\pi(C)\}\right]\, dF(C) \\
&= \int \left[\mathrm{E}(Y^1 - Y^0|X=1, C)\frac{\pi(C)\{Q_\delta(C) - 1\}}{Q_\delta(C) - \pi(C)} + \mathrm{E}(Y^1 - Y^0|X=0, C)\frac{\{1-\pi(C)\}Q_\delta(C)}{Q_\delta(C) - \pi(C)}\right]\{Q_\delta(C) - \pi(C)\}\, dF(C).
\end{aligned}
$$

Therefore, the IPSI causal effect is a weighted average of CACEs across strata defined by the observed exposure X and the measured covariates C. The interior weights are for the CACEs across the two exposure levels, within each stratum defined by the measured covariates C. The exterior weights equal the differences from shifting the current propensity scores to the IPSI.

If we set $\delta = \infty$ so that $Q_{\delta=\infty} = 1$, we can recover the ATC/ATU as:

$$
\begin{aligned}
\mathrm{E}(Y^{Q_{\delta=\infty}}) - \mathrm{E}(Y) &= \int \mathrm{E}(Y^1 - Y^0|X=0, C)\,\{1-\pi(C)\}\, dF(C) \\
&= \Pr(X=0) \int \mathrm{E}(Y^1 - Y^0|X=0, C)\, dF(C|X=0) \\
&= \Pr(X=0)\, \mathrm{E}(Y^1 - Y^0|X=0) \\
\text{ATC/ATU} &= \mathrm{E}(Y^1 - Y^0|X=0) \\
&= \frac{1}{\Pr(X=0)}\left[\mathrm{E}(Y^{Q_{\delta=\infty}}) - \mathrm{E}(Y)\right].
\end{aligned}
$$

Conversely, if we set $\delta = 0$ so that $Q_{\delta=0} = 0$, we can recover the ATT as:

$$
\begin{aligned}
\mathrm{E}(Y^{Q_{\delta=0}}) - \mathrm{E}(Y) &= \int \mathrm{E}(Y^1 - Y^0|X=1, C)\,\{-\pi(C)\}\, dF(C) \\
&= -\Pr(X=1) \int \mathrm{E}(Y^1 - Y^0|X=1, C)\, dF(C|X=1) \\
&= -\Pr(X=1)\, \mathrm{E}(Y^1 - Y^0|X=1) \\
\text{ATT} &= \mathrm{E}(Y^1 - Y^0|X=1) \\
&= \frac{1}{\Pr(X=1)}\left[\mathrm{E}(Y) - \mathrm{E}(Y^{Q_{\delta=0}})\right].
\end{aligned}
$$

Note that these equivalences require only causal or counterfactual consistency.

If we further assume no unmeasured confounding (4) in the main text, then the IPSI causal effect simplifies to:

$$\mathrm{E}(Y^{Q_\delta}) - \mathrm{E}(Y) = \int \underbrace{\mathrm{E}(Y^1 - Y^0 | C)}_{\text{CACE}} \{Q_\delta(C) - \pi(C)\}\; dF(C).$$

Therefore, it is a weighted average of CACEs across strata defined by the measured covariates C, with weights equal to the differences from shifting the current propensity scores to the IPSI. Because the IPSI causal effect depends on the prevailing exposure conditions, as represented by the current propensity scores $\pi(C)$, it may be possible for the shift in δ to be so small as to render the IPSI too small to be detectable, notwithstanding the individual CACEs being non-null.[1]

[1] We thank anonymous reviewer 2 for encouraging us to elaborate on this point.

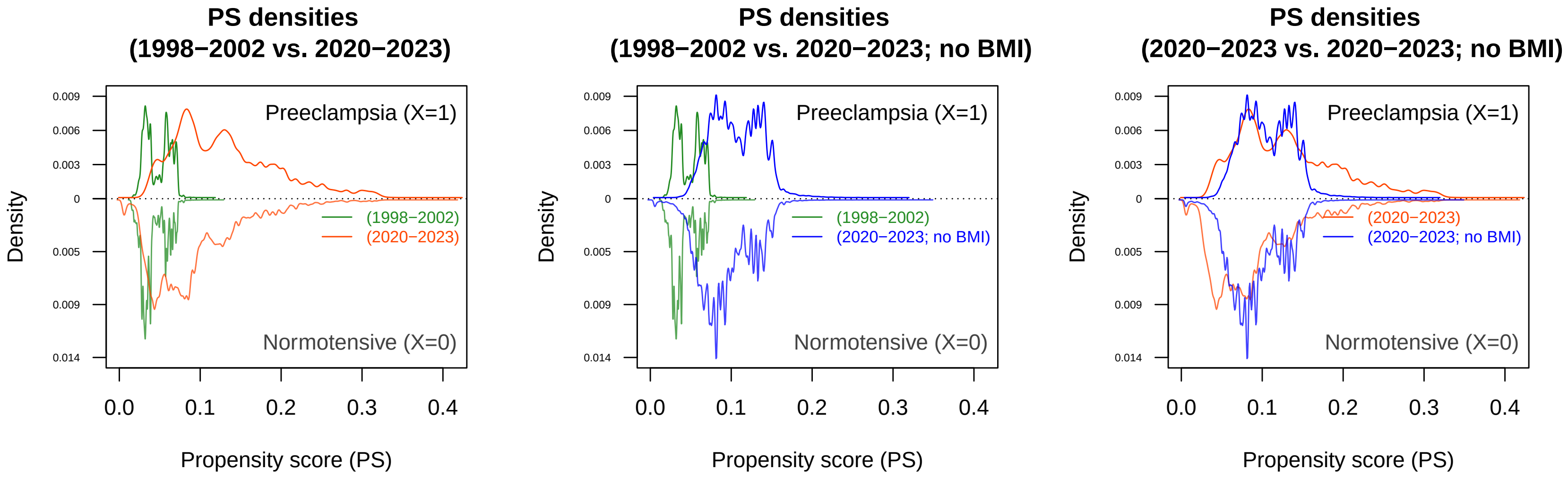

Figure S1: Densities of the estimated (naturally-occurring) propensity scores among gestational hypertensive (top half) and normotensive (bottom half) singleton United States (US) births in 1998-2002 (green) and 2020-2023 (red). For the latter, the PS adjusted for body mass index (BMI) are in red and not adjusted for BMI are in blue. To distinguish the densities visually, the results are shifted slightly away from zero (indicated by the dotted line) on the vertical axis.

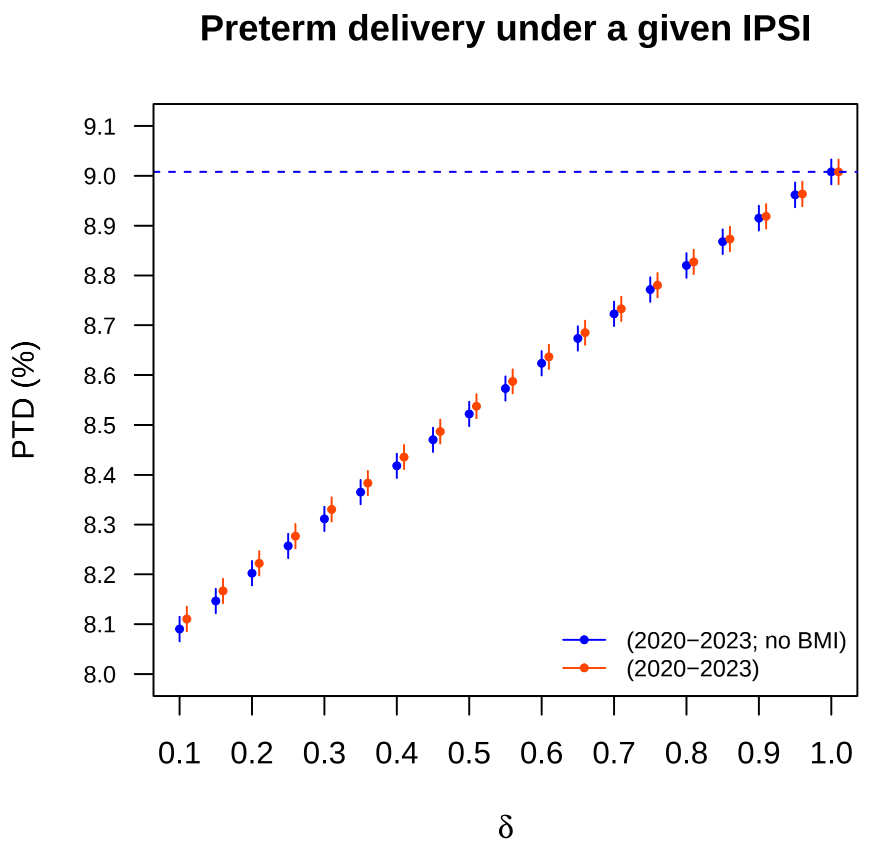

Figure S2: Estimates of the incremental propensity score intervention (IPSI) average preterm delivery (PTD) under different IPSIs encoded by δ using United States (US) singleton births in 2020-2023. The results were based on either adjusting for body mass index (BMI) in the propensity score and outcome models (red) or omitting BMI from those models (blue). Explanations of how to interpret this panel are provided in the caption of Figure 2.

B Sensitivity analysis for IPSI using bias correction factors

In this section, we describe the proposed sensitivity analysis procedure specific to IPSI for putative violations of the unmeasured confounding assumption in (4) of the main text. Assume that supplementing C with an unmeasured (set of) confounder(s) U in the adjustment set suffices for conditional exchangeability, stated as (5) of the main text, to hold. The bias correction formula for the ACE (Theorem 3 in VanderWeele and Arah[1]) offers a closed-form expression for the conditional average potential outcome $\mathrm{E}(Y^x|C)$ in terms of an estimator $\hat{\mathrm{E}}(Y^x|C)$ that does not adjust for U and a bias factor $r_x(c)$ on the risk ratio (RR) scale:

$$\mathrm{E}(Y^x|C) = \frac{\hat{\mathrm{E}}(Y^x|C)}{r_x(C)}, \quad r_x(c) = \frac{\sum_u \gamma_{xcu} \Pr(U = u|X = x, C = c)}{\sum_u \gamma_{xcu} \Pr(U = u|C = c)}.$$

The sensitivity parameter $\gamma_{xcu} = \dfrac{\mathrm{E}(Y|X = x, C = c, U = u)}{\mathrm{E}(Y|X = x, C = c, U = 0)}$ encodes the unmeasured confounder's effect on the RR scale, conditional on X and C, relative to a selected reference level $U = 0$.[1] This general expression allows for binary or multi-valued U, comprising either a single variable or a set of variables.

The joint sensitivity parameters γ_{xcu} and $\Pr(U = u|X = x, C = c)$ must therefore be fixed to specific values to calculate the bias factors $r_x(C), x = 0, 1$, for each individual with observed C. These bias factors are then applied to $\hat{\mathrm{E}}(Y^x|C)$, the predicted potential outcomes using only C, to recover the adjusted potential outcomes accounting for U, e.g., $\tilde{\mathrm{E}}(Y^x|C) = \hat{\mathrm{E}}(Y^x|C)/r_x(C)$. The bias factors are also applied to $\hat{\pi}(C)$, the propensity score estimates using only C, to recover the adjusted propensity score, e.g., $\tilde{\pi}(C) = r_1(C)\hat{\pi}(C)$. The latter follows from the unbiasedness of the inverse probability weighting (IPW) estimator:

$$\mathrm{E}(Y^x|C) = \frac{\hat{\mathrm{E}}(Y^x|C)}{r_x(C)} = \frac{1}{r_x(C)} \mathrm{E}\left[\left. \frac{\mathbb{1}(X = x)\, Y}{\hat{\Pr}(X = x|C)} \right| C \right] = \mathrm{E}\left[\left. \frac{\mathbb{1}(X = x)\, Y}{r_x(C)\hat{\Pr}(X = x|C)} \right| C \right]. \tag{S1}$$

In other words, for those with $X = 1$, the inverse weight would be $r_1(C)\hat{\pi}(C)$, whereas for those with $X = 0$, the inverse weight would be $r_0(C)\{1 - \hat{\pi}(C)\}$. To ensure that the probabilities sum to one, i.e., $r_0(C)\{1 - \hat{\pi}(C)\} + r_1(C)\hat{\pi}(C) = 1$, we must assume the effect of U on Y (on the RR scale) is not modified by X so that $\gamma_{1cu} = \gamma_{0cu}$ for all values of (c, u). This ensures that:

$$\begin{aligned}
&\frac{1 - r_1(c)\hat{\pi}(c)}{1 - \hat{\pi}(c)} \\
&= \frac{\sum_u \gamma_{1cu} \Pr(U = u|C = c) - \hat{\pi}(c) \sum_u \gamma_{1cu} \Pr(U = u|X = 1, C = c)}{\{1 - \hat{\pi}(c)\} \sum_u \gamma_{1cu} \Pr(U = u|C = c)} \\
&= \frac{\sum_u \gamma_{1cu} \Pr(U = u|X = 0, C = c)}{\sum_u \gamma_{1cu} \Pr(U = u|C = c)} \\
&= r_0(c).
\end{aligned}$$

The ensuing IPSI average outcome in (2), having accounted for unmeasured U using the bias factors under the fixed values of the sensitivity parameters, can be identified using $\tilde{\mathrm{E}}(Y^x|C), x = 0, 1$, and $\tilde{\pi}(C)$ as:

$$
\begin{aligned}
\mathrm{E}(Y^{Q_\delta}) &= \int \left[\mathrm{E}(Y^1|C)Q_\delta(C) + \mathrm{E}(Y^0|C)\{1 - Q_\delta(C)\}\right] dF(C) \\
&= \int \left[\frac{\hat{\mathrm{E}}(Y^1|C)}{r_1(C)} \frac{\delta r_1(C)\pi(C)}{1 + (\delta - 1)r_1(C)\pi(C)} + \frac{\hat{\mathrm{E}}(Y^0|C)}{r_0(C)} \left\{1 - \frac{\delta r_1(C)\pi(C)}{1 + (\delta - 1)r_1(C)\pi(C)}\right\}\right] dF(C) \\
&= \int \left[\hat{\mathrm{E}}(Y^1|C) \frac{\delta \pi(C)}{1 + (\delta - 1)r_1(C)\pi(C)} + \hat{\mathrm{E}}(Y^0|C) \left\{\frac{1 - \pi(C)}{1 + (\delta - 1)r_1(C)\pi(C)}\right\}\right] dF(C).
\end{aligned}
$$

B.1 Assumptions for using bias factors

When the no unmeasured confounding assumption holds, positivity (conditional on observed covariates) is unnecessary for IPSI. But when no unmeasured confounding is violated, identification of IPSI effects using bias factors requires the bias factors $r_x(c), x = 0, 1$, to be non-zero for all $C = c$. Hence, allowing for unmeasured confounding introduces positivity (conditional on observed and unobserved covariates) for a sensitivity analysis. Below, we formalize the positivity assumptions for using bias factors.

As stated in (8) and (9), respectively, in the main text, for all $X = x$ and $C = c$,

$$
\begin{gathered}
\Pr(X = x|C = c, U = 0) > 0, \\
\Pr(Y = 1|X = x, C = c, U = 0) > 0
\end{gathered}
$$

The first condition suffices for $\Pr(X = x, C = c, U = 0) = \Pr(X = x|C = c, U = 0) \Pr(U = 0|C = c) \Pr(C = c) > 0$ so that the conditional mean outcome is defined under $U = 0$. Moreover, (8) implies that positivity[2,3] conditional on the observed covariates, stated as (7) in the main text, will hold, because $\Pr(X = x|C = c) = \sum_u \Pr(X = x|C = c, U = u) \Pr(U = u|C = c) > 0$. The second condition ensures γ_{xcu} takes on finite values under the reference level $U = 0$.

To see why both conditions suffice for $r_x(c)$ to be non-zero, write the numerator of $r_x(c)$ as:

$$
\begin{aligned}
&\sum_u \gamma_{xcu} \Pr(U = u|X = x, C = c) \\
&= \gamma_{xcu^*} \Pr(U = u^*|X = x, C = c) + \sum_{u \neq u^*} \gamma_{xcu} \Pr(U = u|X = x, C = c) \\
&= \gamma_{xcu^*} \left\{ 1 - \sum_{u \neq u^*} \Pr(U = u|X = x, C = c) \right\} + \sum_{u \neq u^*} \gamma_{xcu} \Pr(U = u|X = x, C = c) \\
&= \gamma_{xcu^*} + \sum_{u \neq u^*} (\gamma_{xcu} - \gamma_{xcu^*}) \Pr(U = u|X = x, C = c) \\
&= \gamma_{xcu^*} + \sum_{u \neq u^*} (\gamma_{xcu} - \gamma_{xcu^*}) \frac{\Pr(X = x|C = c, U = u) \Pr(U = u|C = c)}{\Pr(X = x|C = c)}.
\end{aligned}
$$

Because $\Pr(X = x|C = c) > 0$ under (8), and setting $u^* = 0$ so that $\gamma_{xcu^*} = 1$ under (9), it follows that $r_x(c) \neq 0$.

Certain violations of the positivity assumption (8) can lead to non-zero values of $r_x(c)$, but there may not be any useful information for a sensitivity analysis. For example, let $\Pr(X = x|C = c, U = u) = \mathbb{1}(u = 0)$, so that $\Pr(X = 1 - x|C = c, U = 0) = 0$ thus violating (8), and $\Pr(U = 0|C = c) = 1$ so that $\Pr(X = x|C = c) = 1$. Then for all $C = c$,

$$
r_x(c) = \frac{\gamma_{xc0} + \sum_{u \neq 0} (\gamma_{xcu} - \gamma_{xc0}) \dfrac{\Pr(X = x|C = c, U = u) \Pr(U = u|C = c)}{\Pr(X = x|C = c)}}{\Pr(U = 0|C = c) + \sum_{u \neq 0} \gamma_{xcu} \Pr(U = u|C = c)} = \frac{\gamma_{xc0}}{\Pr(U = 0|C = c)} = 1.
$$

Hence, when assuming no unmeasured confounding, positivity (conditional on observed covariates) is unnecessary for IPSI. However, when no unmeasured confounding is violated, positivity (conditional on observed and unobserved covariates) is required to use these bias factors for a sensitivity analysis.

B.2 Practical implementation

Unlike current methods using bias correction formulas for other causal effect estimands[4], it is not straightforward under the IPSI approach to practically gauge how the average outcomes simultaneously change over δ and systematically vary over the sensitivity parameters. Therefore, we simplify assumptions in line with those previously applied for the ACE[1] to facilitate reasonably calculating the bias formulas and coherently presenting the sensitivity analysis results in practice.

The first assumes U to be binary. The second assumes a constant effect of U on Y (on the RR scale) across strata of X and C so that $\gamma = \gamma_{xc1}$ for all values of (x, c). Denote the association between U and X

given C by $\nu_x(c) = \Pr(U = 1|X = x, C = c)$. The bias factor then simplifies to:

$$r_x(c) = \frac{1 + (\gamma - 1)\nu_x(c)}{1 + (\gamma - 1)[\pi(c)\nu_1(c) + \{1 - \pi(c)\}\nu_0(c)]},$$

so that

$$r_0(c)^{-1} = 1 + \frac{(\gamma - 1)\{\nu_1(c) - \nu_0(c)\}\pi(c)}{1 + (\gamma - 1)\nu_0(c)}, \quad r_1(c)^{-1} = 1 + \frac{(\gamma - 1)\{\nu_0(c) - \nu_1(c)\}\{1 - \pi(c)\}}{1 + (\gamma - 1)\nu_1(c)}. \tag{S2}$$

Therefore, a larger difference between $\nu_1(c)$ and ν_0(c) encodes a stronger association between U and X that leads to larger transformations of the conditional outcomes using the bias factors.

We propose the following strategy to determine candidate values for the sensitivity parameters using the observed data to plug into the bias factors. The goal is to find values that will yield a marginal causal RR $\frac{\mathrm{E}(Y^1)}{\mathrm{E}(Y^0)}$ as close to one as possible.

1. Calculate the prevalence of each binary measured covariate C_k within each stratum defined by X, i.e., $\Pr(C_k = 1|X = x), x = 0, 1$. These can also include interactions between the covariates, e.g., for $j \neq k, \Pr(C_j C_k = 1|X = x), x = 0, 1$. Let the minimum and maximum empirical values of the observed covariates within each exposure level $X = x$ be denoted by:

$$\begin{aligned}
\nu_x^{\min} &= \min\left\{\min_k \Pr(C_k = 1|X = x), \min_{j\neq k, j<k} \Pr(C_j C_k = 1|X = x)\right\},\\
\nu_x^{\max} &= \max\left\{\max_k \Pr(C_k = 1|X = x), \max_{j\neq k, j<k} \Pr(C_j C_k = 1|X = x)\right\}.
\end{aligned}$$

2. Set $\nu_1(c) = \nu_1^{\min}, \nu_0(c) = \nu_0^{\max}$. For the moment, we have assumed for $x = 0, 1$, that $\nu_x = \nu_x(c)$ for all c. We describe later how to relax this assumption.

3. Postulate a discrete grid of values for γ. For simplicity, we used 50 equally spaced values on the natural logarithmic scale between 1 and $\exp(-4)$ or $\exp(4)$ for 101 distinct values.

4. Calculate the bias factors in (S2) for each postulated value of γ in step 3 with the fixed values of $\nu_x(c), x = 0, 1$, from step 2. Use the bias factors to estimate the marginal causal RR:

$$\frac{\mathrm{E}(Y^1)}{\mathrm{E}(Y^0)} = \frac{\int \mathrm{E}\left[\left.\frac{\mathbb{1}(X = 1)\,Y}{r_1(C)\hat{\pi}(C)}\right| C\right] dF(C)}{\int \mathrm{E}\left[\left.\frac{\mathbb{1}(X = 0)\,Y}{r_0(C)\{1 - \hat{\pi}(C)\}}\right| C\right] dF(C)}. \tag{S3}$$

We elected to use the IPW estimator to emphasize the IPSI's reliance on the propensity scores. Bias

factors that lead to adjusted propensity score values close to zero or one can result in large weights, indicating potential violations of positivity.[5]

5. Select the value of γ that yields a marginal causal risk ratio closest to one.

6. Use the fixed values of $\{\gamma, \nu_1(c), \nu_0(c)\}$ to calculate the bias factors, obtain the adjusted potential outcomes and propensity scores, and reestimate the IPSI average outcomes under the posited values of δ.

The estimated RRs in (S3) as γ varied following step 4 are plotted in the leftmost panels of the top rows in Figures S3 and S4 for each cohort.

The sensitivity parameters $\{\nu_1(c), \nu_0(c)\}$ can also be fixed at different values in step 2. For example, we could set $\nu_1(c) = \nu_1^{\max}, \nu_0(c) = \nu_0^{\min}$. The RRs as a function of γ following step 4 for each cohort, under these fixed values of $\{\nu_1(c), \nu_0(c)\}$ in step 2, are plotted in the center-left panels of the top row in Figures S3 and S4 for each cohort. To relax the assumption that the association between U and X does not depend on C, i.e., $\nu_x = \nu_x(C)$, we propose allowing $\nu_x(C)$ to differ across C by way of $\pi(C)$. Specifically, we seek to shift the adjusted propensity score $\tilde{\pi}(C)$ to be smaller if $\hat{\pi}(C) < \hat{\mathrm{E}}(X)$, or larger if $\hat{\pi}(C) \geq \hat{\mathrm{E}}(X)$; i.e.,

$$\nu_1(c) = \begin{cases} \nu_1^{\min}, & \hat{\pi}(C) < \hat{\mathrm{E}}(X) \\ \nu_1^{\max}, & \hat{\pi}(C) \geq \hat{\mathrm{E}}(X) \end{cases} \quad ; \quad \nu_0(c) = \begin{cases} \nu_0^{\max}, & \hat{\pi}(C) < \hat{\mathrm{E}}(X) \\ \nu_0^{\min}, & \hat{\pi}(C) \geq \hat{\mathrm{E}}(X) \end{cases} .$$

For completeness, another possibility is to swap the minimum and maximum values; i.e.,

$$\nu_1(c) = \begin{cases} \nu_1^{\max}, & \hat{\pi}(C) < \hat{\mathrm{E}}(X) \\ \nu_1^{\min}, & \hat{\pi}(C) \geq \hat{\mathrm{E}}(X) \end{cases} \quad ; \quad \nu_0(c) = \begin{cases} \nu_0^{\min}, & \hat{\pi}(C) < \hat{\mathrm{E}}(X) \\ \nu_0^{\max}, & \hat{\pi}(C) \geq \hat{\mathrm{E}}(X) \end{cases} .$$

The RRs as a function of γ following step 4 for each cohort, under these fixed values of $\{\nu_1(c), \nu_0(c)\}$ in step 2, are plotted in the center-right and rightmost panels, respectively, of the top row in Figures S3 and S4 for each cohort.

The IPSI average outcomes under the different posited values of δ following step 6 are plotted in the bottom rows of Figures S3 and S4 for each cohort. Each panel corresponds to the fixed values of the sensitivity parameters shown in the panel above. In all considered scenarios, the IPSI average outcomes decreased with δ at only very slightly different rates depending on the fixed sensitivity parameter values.

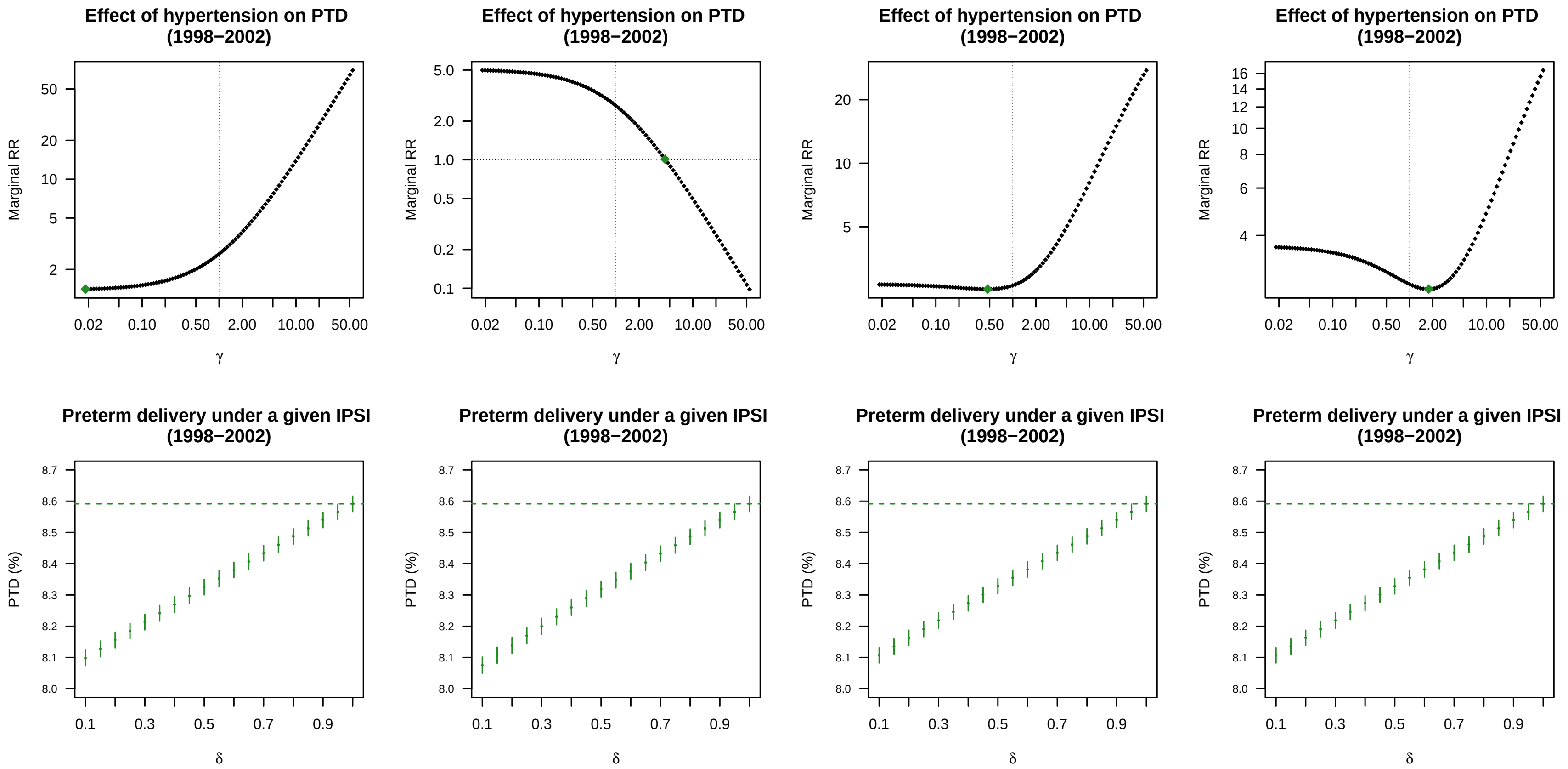

Figure S3: Estimates of the marginal causal risk ratio (RR) of preterm delivery (PTD) between preeclamptic and normotensive births (top row) and the incremental propensity score (IPSI) average PTD (%; bottom row) using United States (US) singleton births in 1998-2002, following a sensitivity analysis for unmeasured confounding. In the top row, each point corresponds to a given sensitivity parameter value γ on the horizontal axis. The (larger) point in green corresponds to the value of γ that yielded the RR estimate closest to one. Each column corresponds to a fixed value of the sensitivity parameters $\{\nu_1(c), \nu_0(c)\}$ as described in the Supplemental material. Details about the interpretation of the panels in the bottom row are described in the caption of Figure 2 of the main text.

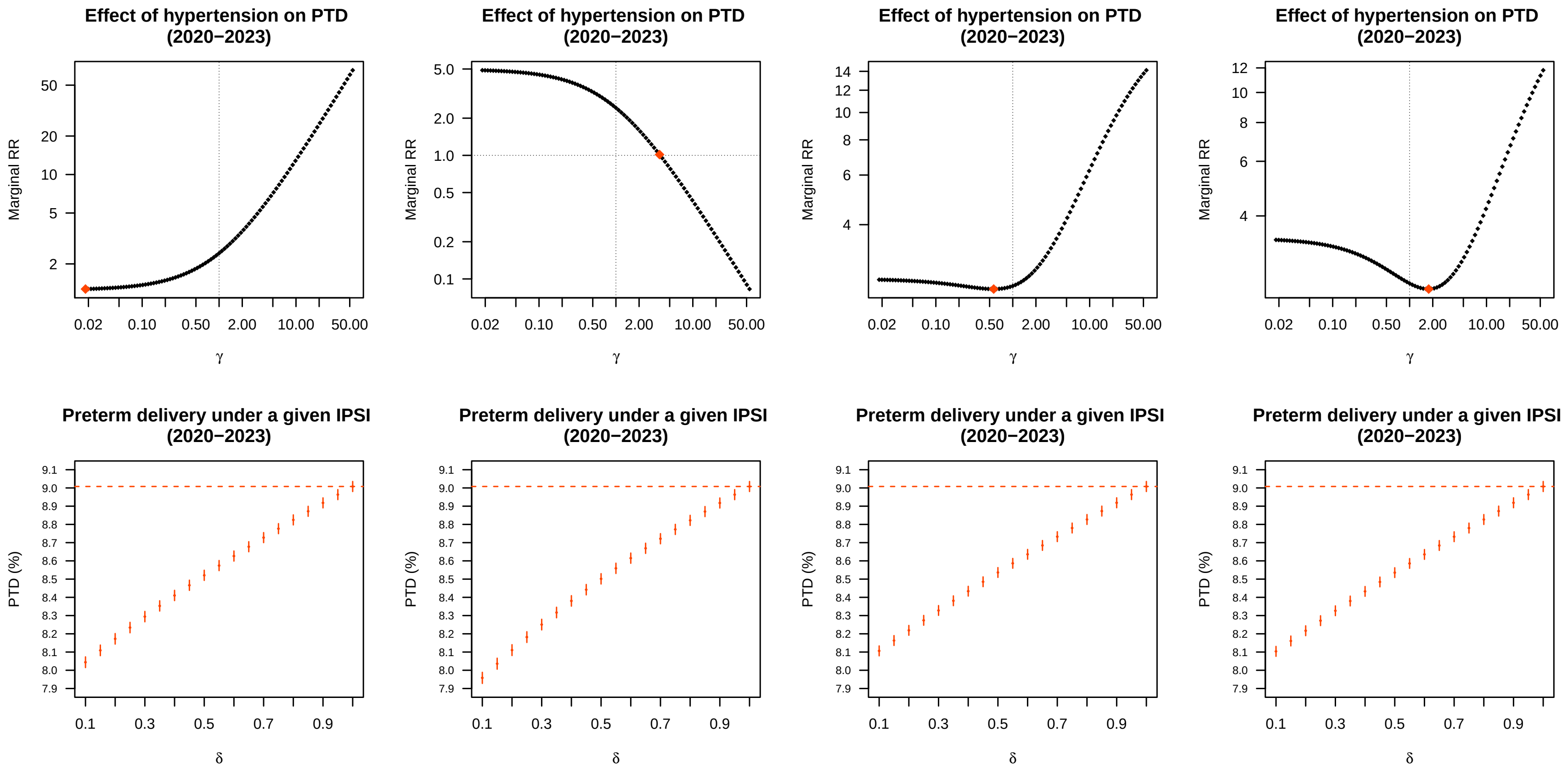

Figure S4: Estimates of the marginal causal risk ratio (RR) of preterm delivery (PTD) between preeclamptic and normotensive births (top row) and the incremental propensity score (IPSI) average PTD (%; bottom row) using United States (US) singleton births in 2020-2023, following a sensitivity analysis for unmeasured confounding. In the top row, each point corresponds to a given sensitivity parameter value γ on the horizontal axis. The (larger) point in red corresponds to the value of γ that yielded the RR estimate closest to one. Each column corresponds to a fixed value of the sensitivity parameters $\{\nu_1(c), \nu_0(c)\}$ as described in the Supplemental material. Details about the interpretation of the panels in the bottom row are described in the caption of Figure 2 of the main text.

References

1. VanderWeele TJ and Arah OA. Bias formulas for sensitivity analysis of unmeasured confounding for general outcomes, treatments, and confounders. Epidemiology 2011; 22:42–52

2. Petersen ML, Porter KE, Gruber S, Wang Y, and Van Der Laan MJ. Diagnosing and responding to violations in the positivity assumption. Statistical methods in medical research 2012; 21:31–54

3. Westreich D and Cole SR. Invited Commentary: Positivity in Practice. American Journal of Epidemiology 2010; 171:674–7. DOI: 10.1093/aje/kwp436

4. Loh WW and Ananth CV. Does Adjusting for Causal Intermediate Confounders Resolve the Perinatal Crossover Paradox? Epidemiology 2025; 36. DOI: 10.1097/EDE.0000000000001848

5. Bonvini M, McClean A, Branson Z, and Kennedy EH. Incremental Causal Effects: An Introduction and Review. *Handbook of Matching and Weighting Adjustments for Causal Inference*. Ed. by Zubizarreta JR, Stuart EA, Small DS, and Rosenbaum PR. 1st. Chapman and Hall/CRC, 2023 :349–72. DOI: 10.1201/9781003102670
